# Regularized Multidirections and Multiscales Anisotropic Diffusion for Sinogram Restoration of Low-Dosed Computed Tomography

**DOI:** 10.1155/2013/190571

**Published:** 2013-11-21

**Authors:** Zhiwu Liao

**Affiliations:** School of Computer Science, Sichuan Normal University, Chengdu, Sichuan 610101, China

## Abstract

Although most of existing anisotropic diffusion (AD) methods are supported by prefect mathematical theories, they still lead to smoothed edges and anatomy details (EADs). They are caused by not considering the discrete nature of digital signal. In order to improve the performance of AD in sinogram restoration of low-dosed computed tomography (LDCT), we propose a new AD method, named regularized multidirections and multiscales anisotropic diffusion (RMDMS-AD), by extending AD to regularized AD (RAD) in multidirections and multiscales. Since the multidirections can reduce the discrete errors to the maximum extent, meanwhile multiscales and RAD make searching neighborhood of solution be as large as possible which can get more optimal solution to AD, the new proposed method can improve the performance of AD both in denoising and in stability of solution. Moreover, the discrete errors and ill-posed solutions occur mostly near the EADs; the RMDMS-AD will also preserve EADs well. Comparing the proposed new method to existing AD methods using real sinogram, the new method shows good performance in EADs preserving while denoising and suppressing artifacts.

## 1. Introduction

Anisotropic diffusion (AD) proposed in 1990, also called Perona-Malik diffusion (PMD), is a technique aiming at reducing image details without removing significant parts of the image contents, typically edges, lines, or textures which are important for the image [1]. Formally, AD is defined as

(1) $$\begin{array}{c} \;\frac{\partial u\left(x,y,t\right)}{\partial t}=\mathrm{div}\left(g\left(x,y,t\right)\nabla u\left(x,y,t\right)\right), \end{array}$$

where *u*(*x*, *y*, 0) is the initial gray scale image, *u*(*x*, *y*, *t*) is the smoothed gray scale image at time *t*, ∇ denotes the gradient, div⁡(·) is the divergence operator, and *g*(*x*, *y*, *t*) is the diffusion coefficient. *g*(*x*, *y*, *t*) controls the rate of diffusion and is usually chosen as a monotonically decreasing function of the module of the image gradient. The coefficients usually take the following two forms:

(2) $$\begin{array}{c} g\left(||\nabla u\left(x,y,t\right)||\right)=e^{-{\left(||\nabla u(x,y,t)||/\sigma \right)}^{2}}, \end{array}$$

(3) $$\begin{array}{c} g\left(||\nabla u\left(x,y,t\right)||\right)=\frac{1}{1+{\left(||\nabla u\left(x,y,t\right)||/\sigma \right)}^{2}}, \end{array}$$

where ||·|| is the module of the vector and the constant *σ* controls the sensitivity to edges.

The key idea of PMD is to smooth the homogenous regions with small ||∇*u*(*x*, *y*, *t*)|| while, near singularities with big ||∇*u*(*x*, *y*, *t*)||, PMD is only smootheed along the perpendicular direction of the gradient.

So far, much research has been devoted to improving the performance of PMD from the view of mathematical theory [2–11]. In 1992, Catté et al. indicate that PMD is ill-posed, and they also propose a new well-posed method named regularized anisotropic diffusion (RAD) [2].

Some recent efforts also focus on the illness of PMD and propose that posing some conditions on diffusion coefficients will make the PMD become well posed [3–6]. These conditions include posing edge influence functions near edges [3] and substituting the module of the gradient operator to a nearby differentiable function [6].

Since the main idea for AD is that diffusion should be along the normal near singularity, how to approximate to directions of gradient and normal (DsGN) in discrete data turns very important in designing AD filtering [1, 2]. From the coherence filtering proposed by Weickert [8] in 1999, vector tensor becomes a preferable alternative for DsGN since the tensor not only can be extended to high dimensional data directly but also can avoid computing DsGN [9–11].

Essentially, tensor is also a 2-dimension method for data analysis, which can not provide enough directions for diffusing in discrete situation. Thus, diffusion according to tensor will also make serious artifacts, which hampers AD to be used for low-dose CT imaging.

Recently, how to minimize the radiation exposure to patients has been one of the major efforts in modern clinical X-ray CT radiology because of the associated risk of cancer for patients receiving CT examination [12–16]. But LDCT results in the presentation of strong noise and artifacts which will degrade the quality of LDCT images dramatically and decrease the accuracy of diagnosis.

Filtering noise from clinical scans is a challenging task since these scans contain artifacts and noise and consist of many structures with different shapes, sizes, and contrast, which should be preserved for making correct diagnosis. Many strategies have been proposed to improve the imaging quality of LDCT [17–24]. These methods include some state-of-the-art techniques, such as nonlocal means [18].

Very recently, a new AD, named time and space AD (TSAD), which extends classical space AD to time and space AD by adding an extra diffusion dimension of time, is proposed and shows promising potential in preserving singularities of LDCT scans while denoising [25]. Because much more neighbor pixels will be used in searching solution of *u*(*x*, *y*), TSAD can obtain more optimal solution than traditional AD and RAD.

However, the searching neighborhood of TSAD is still not big enough especially near singularities, which will blur the edges. Therefore, how to expand searching neighborhood for resolution of AD is a key problem to improve performance of AD. In this paper, we argue that diffusion should be carried on multiscales in multidirections in order to search a solution in larger neighborhood and to reduce discrete errors.

It should be indicated that multiscales in this paper are different to multiscales in multiscale analysis, which used one scale in a time while, in this paper, multiscales are used simultaneously to extend one scale searching neighborhood, whose details will be introduced in Section 3. The proposed method also is different from RPM since RPM only regularizes diffusion coefficients while our method provides more pixels for diffusion itself.

The arrangement of this paper is as follows. In Section 2, the backgrounds about noise model of LDCT and AD are introduced; and then the new diffusion method is given in Section 3; the experiment results are shown and discussed in Section 4; the final part is the conclusions and acknowledgment.

## 2. Backgrounds

In this section some related backgrounds will be introduced. These backgrounds include noise models and numerical schemes for AD and RAD. 

### 2.1. Noise Model

Poisson noise (PN) and Gaussian dependent noise (GDN) after some transform of original sinogram are generally accepted as two noise models of LDCT [26, 27]. These two models are built both on repeated phantom experiments and theory analysis.

The photon noise is due to the limited number of photons collected by the detector [27]. For a given attenuating path in the imaged subject, *N*_0_(*i*, *α*) and *N*(*i*, *α*) denote the incident and the penetrated photon numbers, respectively. Here, *i* denotes the index of detector channel or bin and *α* is the index of projection angle. In the presence of noises, the sinogram should be considered as a random process and the attenuating path is given by

(4) $$\begin{array}{c} r_{i}=-\ln\left[\frac{N\left(i,\alpha \right)}{N_{0}\left(i,\alpha \right)}\right], \end{array}$$

where *N*_0_(*i*, *α*) is a constant and *N*(*i*, *α*) is Poisson distribution with mean *N*.

Thus, we have

(5) $$\begin{array}{c} N\left(i,\alpha \right)=N_{0}\left(i,\alpha \right)\exp\left(-r_{i}\right). \end{array}$$

Both its mean value and variance are *N*.

Gaussian distributions of ployenergetic systems were assumed based on limited theorem for high-flux levels, and followed by many repeated experiments in [26], we have

(6) $$\begin{array}{c} \sigma_{i}^{2}\left(\mu_{i}\right)=f_{i}\exp\left(\frac{\mu_{i}}{\gamma }\right), \end{array}$$

where *μ*_*i*_ is the mean and *σ*_*i*_^2^ is the variance of the projection data at detector channel or bin *i*, *γ* is a scaling parameter, and *f*_*i*_ is a parameter adaptive to different detector bins.

The most common conclusion for the relation between Poisson distribution and Gaussian distribution is that the photon count will obey Gaussian distribution for the case with large incident intensity and Poisson distribution with feeble intensity [27]. In addition, in [26], the authors deduce the equivalency between Poisson model and Gaussian model. Therefore, both theories indicate that these two noises have similar statistical properties and can be unified into a whole framework.

### 2.2. The Numerical Schemes for PM and RPM

In Section 1, AD is defined as in (1)–(3), which encourage diffusion (hence smoothing) within regions and stop it near strong edges. Hence the edges can be preserved while smoothing from the image [1].

The discretization for Laplacian operator is

(7) u(i,j,t+1)  =u(i,j,t)+14[cN·∇N2u(i,j,t)           +cS·∇S2u(i,j,t)           +cE·∇E2u(i,j,t)           +cW·∇W2u(i,j,t)],

where

(8) $$\begin{array}{c} \nabla_{N}^{2}u\left(i,j,t\right)=u\left(i-1,j,t\right)-u\left(i,j,t\right),\\ \nabla_{S}^{2}u\left(i,j,t\right)=u\left(i+1,j,t\right)-u\left(i,j,t\right),\\ \nabla_{E}^{2}u\left(i,j,t\right)=u\left(i,j+1,t\right)-u\left(i,j,t\right),\\ \nabla_{W}^{2}u\left(i,j,t\right)=u\left(i,j-1,t\right)-u\left(i,j,t\right). \end{array}$$

According to (1)–(3), the diffusion coefficient is defined as a function of module of the gradient. However, computing a gradient accurately in discrete data is very complex and the module of the gradient is simplified as the absolute values of four directions and diffusion coefficients are

(9) $$\begin{array}{c} c_{N}\left(i,j,t\right)=g\left(\left|\nabla_{N}^{2}u\left(i,j,t\right)\right|\right),\\ c_{S}\left(i,j,t\right)=g\left(\left|\nabla_{S}^{2}u\left(i,j,t\right)\right|\right),\\ c_{E}\left(i,j,t\right)=g\left(\left|\nabla_{E}^{2}u\left(i,j,t\right)\right|\right),\\ c_{W}\left(i,j,t\right)=g\left(\left|\nabla_{W}^{2}u\left(i,j,t\right)\right|\right), \end{array}$$

where ||·|| is the absolute value of the number and *g*(·) is defined in (2) or (3).

However, PMD is an ill-posed equation and it can be well posed by RAD [2], which is defined as

(10) $$\begin{array}{c} \frac{\partial u\left(x,y,t\right)}{\partial t}=\mathrm{div}\left(g\left(||G_{\sigma_{1}}\cdot \nabla u\left(x,y,t\right)||\right)\nabla u\left(x,y,t\right)\right). \end{array}$$

Here *G*_*σ*_1__ defined as

(11) $$\begin{array}{c} G_{\sigma_{1}}=\frac{1}{C}e^{-\left(x^{2}+y^{2}\right)/\sigma_{1}^{2}} \end{array}$$

is a Gaussian function and *C* is a constant. The diffusion coefficients *g*(·) are defined in (2) or (3).

The discretization for Laplacian operator is defined in (7) and (8). But the module of the gradient is simplified as the absolute values of smoothed four directions and diffusion coefficients are

(12) $$\begin{array}{c} c_{N}\left(i,j,t\right)=g\left(\left|G_{\sigma_{1}}\cdot \nabla_{N}^{2}u\left(i,j,t\right)\right|\right),\\ c_{S}\left(i,j,t\right)=g\left(\left|G_{\sigma_{1}}\cdot \nabla_{S}^{2}u\left(i,j,t\right)\right|\right),\\ c_{E}\left(i,j,t\right)=g\left(\left|G_{\sigma_{1}}\cdot \nabla_{E}^{2}u\left(i,j,t\right)\right|\right),\\ c_{W}\left(i,j,t\right)=g\left(\left|G_{\sigma_{1}}\cdot \nabla_{W}^{2}u\left(i,j,t\right)\right|\right), \end{array}$$

where ||·|| is the absolute value of the number, *G*_*σ*_1__· is defined in (11), and *g*(·) is defined in (2) or (3).

## 3. The New Method

In this section, related definitions of multidirections for the first and second scale are given firstly; then the general definitions for the *n*th scale (*n* > 2) are provided. Finally, the new numerical scheme of AD is proposed by mixing the multi scale information together.

### 3.1. Multidirections-Enclosed Difference of the First Scale

**Figure 1:**
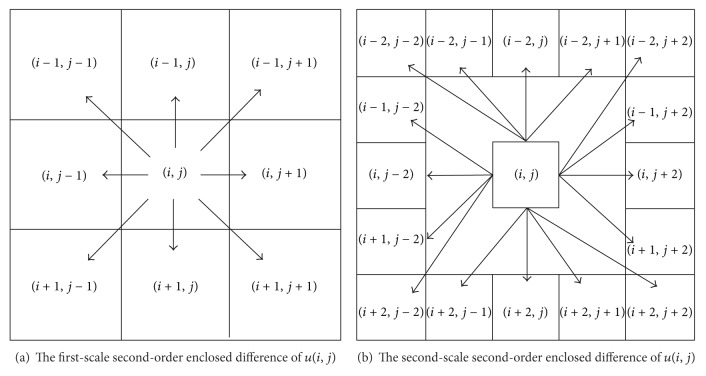
The second-order enclosed difference of *u*(*i*, *j*).

Intuitively, more directions will obtain better performance in image smoothing. In order to extend four directions to the most directions, enclosed directions for *u*(*i*, *j*) which are defined as the differences between *u*(*i*, *j*) and its enclosed nearest neighbors in [28] are adopt. For example, the differences between *u*(*i*, *j*) and its eight-nearest-neighbors are their first-scale enclosed directions (see Figure 1(a)).

Although the motivation of multidirections is very simple, its theory is based on *the high dimensional vector analysis for AD*. The high dimensional vector used in it is half-point differences between the center point *u*(*i*, *j*) and its nearest eight or more half points. The first-scale enclosed gradient vector is defined as

(13) ∇→u(i,j)=(∇0u(i,j),∇1u(i,j), ∇2u(i,j),∇3u(i,j),∇4u(i,j), ∇5u(i,j),∇6u(i,j),∇7u(i,j))T,

where *T* represents the transpose of the vector and ∇*u*_*k*_(*i*, *j*), *k* = 0,…, 7 are defined as

(14) $$\begin{array}{c} \nabla_{0}u\left(i,j\right)=u\left(i,j+0.5\right)-u\left(i,j\right),\\ \nabla_{1}u\left(i,j\right)=u\left(i-0.5,j+0.5\right)-u\left(i,j\right),\\ \nabla_{2}u\left(i,j\right)=u\left(i-0.5,j\right)-u\left(i,j\right),\\ \nabla_{3}u\left(i,j\right)=u\left(i-0.5,j-0.5\right)-u\left(i,j\right),\\ \nabla_{4}u\left(i,j\right)=u\left(i,j-0.5\right)-u\left(i,j\right),\\ \nabla_{5}u\left(i,j\right)=u\left(i+0.5,j-0.5\right)-u\left(i,j\right),\\ \nabla_{6}u\left(i,j\right)=u\left(i+0.5,j\right)-u\left(i,j\right),\\ \nabla_{7}u\left(i,j\right)=u\left(i+0.5,j+0.5\right)-u\left(i,j\right). \end{array}$$

Thus the first-scale second-order difference of *u*(*i*, *j*) is

(15) ∇→2u(i,j)=(∇02u(i,j),∇12u(i,j), ∇22u(i,j),∇32u(i,j),∇42u(i,j), ∇52u(i,j),∇62u(i,j),∇72u(i,j))T,

where *T* represents the transpose of the vector. From (13) we have

(16) $$\begin{array}{c} \nabla_{0}^{2}u\left(i,j\right)=u\left(i,j+1\right)-u\left(i,j\right),\\ \nabla_{1}^{2}u\left(i,j\right)=u\left(i-1,j+1\right)-u\left(i,j\right),\\ \nabla_{2}^{2}u\left(i,j\right)=u\left(i-1,j\right)-u\left(i,j\right),\\ \nabla_{3}^{2}u\left(i,j\right)=u\left(i-1,j-1\right)-u\left(i,j\right),\\ \nabla_{4}^{2}u\left(i,j\right)=u\left(i,j-1\right)-u\left(i,j\right),\\ \nabla_{5}^{2}u\left(i,j\right)=u\left(i+1,j-1\right)-u\left(i,j\right),\\ \nabla_{6}^{2}u\left(i,j\right)=u\left(i+1,j\right)-u\left(i,j\right),\\ \nabla_{7}^{2}u\left(i,j\right)=u\left(i+1,j+1\right)-u\left(i,j\right). \end{array}$$

It can be easily concluded that the first scale second-order difference of *u*(*i*, *j*) is composed by eight components which are the differences between *u*(*i*, *j*) and its enclosed eight-nearest-neighbors. Except for the points on border of an image, each point is enclosed by its eight-nearest-neighbors, so the first-scale second-order difference of *u*(*i*, *j*) is called the enclosed first-scale second-order difference of *u*(*i*, *j*).

**Definition 1**

The first-scale second-order-difference of *u*(*i*, *j*) defined in (15) is called *the first-scale second-order enclosed difference of u*(*i*, *j*).

Based on Definition 1, we have the following.

**Definition 2**

Let the first-scale second-order difference of *u*(*i*, *j*) defined in (15). The following equation

(17) $$\begin{array}{c} \sum_{k=0}^{7}\nabla_{k}^{2}u\left(i,j\right) \end{array}$$

is called *the first-scale enclosed Laplacian operator (ELO) of U*(*i*, *j*).

### 3.2. Multidirections of the *n*th Scale

In this section, multidirections of the second scale are introduced firstly to enhance the intuition of multidirections for the *n*th scale; and then, the related definitions of multidirections of the *n*th scale are given.

Just as shown in Figure 1(b), there are 16-nearest-neighbors in the second scale to form enclosed directions. Thus, the second-scale enclosed gradient vector is defined as

(18) ∇→u(i,j)  =(∇0u(i,j),∇1u(i,j),…,∇15u(i,j))T,

where *T* represents the transpose of the vector and ∇*u*_*k*_(*i*, *j*), *k* = 0,…, 15, are defined as

(19) $$\begin{array}{c} \nabla_{0}u\left(i,j\right)=u\left(i,j+1\right)-u\left(i,j\right),\\ \nabla_{1}u\left(i,j\right)=u\left(i-0.5,j+1\right)-u\left(i,j\right),\\ \nabla_{2}u\left(i,j\right)=u\left(i-1,j+1\right)-u\left(i,j\right),\\ \nabla_{3}u\left(i,j\right)=u\left(i-1,j+0.5\right)-u\left(i,j\right),\\ \vdots \\ \nabla_{14}u\left(i,j\right)=u\left(i+1,j+1\right)-u\left(i,j\right),\\ \nabla_{15}u\left(i,j\right)=u\left(i+0.5,j+1\right)-u\left(i,j\right). \end{array}$$

The second-scale second-order difference of *u*(*i*, *j*) is

(20) $$\begin{array}{c} {\vec{\nabla }}^{2}u\left(i,j\right)={\left(\nabla_{0}^{2}u\left(i,j\right),\nabla_{1}^{2}u\left(i,j\right),\ldots ,\nabla_{15}^{2}u\left(i,j\right)\right)}^{T}, \end{array}$$

where *T* represents the transpose of the vector. We have

(21) $$\begin{array}{c} \nabla_{0}^{2}u\left(i,j\right)=u\left(i,j+2\right)-u\left(i,j\right),\\ \nabla_{1}^{2}u\left(i,j\right)=u\left(i-1,j+2\right)-u\left(i,j\right),\\ \nabla_{2}^{2}u\left(i,j\right)=u\left(i-2,j+2\right)-u\left(i,j\right),\\ \nabla_{3}^{2}u\left(i,j\right)=u\left(i-2,j+1\right)-u\left(i,j\right),\\ \vdots \\ \nabla_{14}^{2}u\left(i,j\right)=u\left(i+2,j+2\right)-u\left(i,j\right),\\ \nabla_{15}^{2}u\left(i,j\right)=u\left(i+1,j+2\right)-u\left(i,j\right). \end{array}$$

Similar to previous discussion, the second-scale second-order difference is defined as follows.

**Definition 3**

The second-scale second-order difference of *u*(*i*, *j*) defined in (20) is called *the second-scale second-order enclosed difference of u*(*i*, *j*).

Based on Definition 3, we have the following.

**Definition 4**

Let the second-scale second-order difference of *u*(*i*, *j*) defined in (20). The following equation

(22) $$\begin{array}{c} \sum_{k=0}^{15}\nabla_{k}^{2}u\left(i,j\right) \end{array}$$

is called *the second-scale enclosed Laplacian operator (ELO) of U*(*i*, *j*).

In order to discuss the general definition of multidirections of the *n*th scale (*n* > 2), we have to determine the number of multidirections for the *n*th scale firstly:

(23) $$\begin{array}{c} {\left(2n+1\right)}^{2}-{\left(2n-1\right)}^{2}=8n. \end{array}$$

Therefore, the *n*th scale enclosed gradient vector is defined as

(24) $$\begin{array}{c} \vec{\nabla }u\left(i,j\right)={\left(\nabla_{0}u\left(i,j\right),\nabla_{1}u\left(i,j\right),\ldots ,\nabla_{8n-1}u\left(i,j\right)\right)}^{T}, \end{array}$$

where *T* represents the transpose of the vector and ∇*u*_*k*_(*i*, *j*), *k* = 0,…, 8*n* − 1, are defined as

(25) $$\begin{array}{c} \nabla_{0}u\left(i,j\right)=u\left(i,j+\frac{n}{2}\right)-u\left(i,j\right),\\ \nabla_{1}u\left(i,j\right)=u\left(i-0.5,j+\frac{n}{2}\right)-u\left(i,j\right),\\ \nabla_{2}u\left(i,j\right)=u\left(i-1,j+\frac{n}{2}\right)-u\left(i,j\right),\\ \nabla_{3}u\left(i,j\right)=u\left(i-1.5,j+\frac{n}{2}\right)-u\left(i,j\right),\\ \vdots \\ \nabla_{8n-2}u\left(i,j\right)=u\left(i+1,j+\frac{n}{2}\right)-u\left(i,j\right),\\ \nabla_{8n-1}u\left(i,j\right)=u\left(i+0.5,j+\frac{n}{2}\right)-u\left(i,j\right). \end{array}$$

The *n*th scale second-order difference of *u*(*i*, *j*) is

(26) $$\begin{array}{c} {\vec{\nabla }}^{2}u\left(i,j\right)={\left(\nabla_{0}^{2}u\left(i,j\right),\nabla_{1}^{2}u\left(i,j\right),\ldots ,\nabla_{8n-1}^{2}u\left(i,j\right)\right)}^{T}, \end{array}$$

where *T* represents the transpose of the vector. We have

(27) $$\begin{array}{c} \nabla_{0}^{2}u\left(i,j\right)=u\left(i,j+n\right)-u\left(i,j\right),\\ \nabla_{1}^{2}u\left(i,j\right)=u\left(i-1,j+n\right)-u\left(i,j\right),\\ \nabla_{2}^{2}u\left(i,j\right)=u\left(i-2,j+n\right)-u\left(i,j\right),\\ \nabla_{3}^{2}u\left(i,j\right)=u\left(i-3,j+n\right)-u\left(i,j\right),\\ \vdots \\ \nabla_{8n-2}^{2}u\left(i,j\right)=u\left(i+2,j+n\right)-u\left(i,j\right),\\ \nabla_{8n-1}^{2}u\left(i,j\right)=u\left(i+1,j+n\right)-u\left(i,j\right). \end{array}$$

The *n*th scale second-order difference is defined as follows.

**Definition 5**

The *n*th scale second-order difference of *u*(*i*, *j*) defined in (26) is called *the* *nth scale second-order enclosed difference of u*(*i*, *j*).

Based on Definition 5, we have the following.

**Definition**

Let the *n*th scale second-order difference of *u*(*i*, *j*) defined in (26). The following equation

(28) $$\begin{array}{c} \sum_{k=0}^{8n-1}\nabla_{k}^{2}u\left(i,j\right) \end{array}$$

is called the *nth scale enclosed Laplacian operator (ELO) of U*(*i*, *j*).

### 3.3. The New Numerical Scheme

Although the form of the PDE is the same as the RAD model equations (10)-(11), the numerical scheme in this paper is quite different from the RAD.

Let

(29) $$\begin{array}{c} \vec{g}={\left(g_{0},g_{1},\ldots ,g_{{\left(2n+1\right)}^{2}-1}\right)}^{T}, \end{array}$$

where *n* > 2 is an integer, *T* represents the transpose of the vector and *g*_*k*_, *k* = 0,…, (2*n* + 1)^2^ − 1, is defined as

(30) $$\begin{array}{c} g_{k}=\frac{g\left(\left|G_{\sigma_{1}}\cdot \nabla_{k}u\left(i,j\right)\right|\right)}{\sum_{t=0}^{{\left(2n+1\right)}^{2}-1}g\left(\left|G_{\sigma_{1}}\cdot \nabla_{t}u\left(i,j\right)\right|\right)}, \end{array}$$

where *G*_*σ*_1__ is defined in (11), ∇_*k*_ *u*(*i*, *j*), *k* = 0,…, (2*n* + 1)^2^ − 1, defined in (24) are the components of vector $\vec{\nabla }u(i,j)$, ∑_*t*=0_^(2*n*+1)^2^−1^ *g*(|*G*_*σ*_1__ · ∇_*t*_ *u*(*i*, *j*)|) is the normalized constant, and *g* is the decreasing function of absolute value of ∇_*k*_ *u*(*i*, *j*), *k* = 0,…, (2*n* + 1)^2^ − 1. Following (2) and (3), *g*(|*G*_*σ*_1__ · ∇*u*_*k*_(*x*, *y*, *t*)|) can be defined as

(31) $$\begin{array}{c} g\left(\left|G_{\sigma_{1}}\cdot \nabla u_{k}\left(x,y,t\right)\right|\right)=e^{-{\left(|G_{\sigma_{1}}\cdot \nabla u_{k}(x,y,t)|/\sigma \right)}^{2}} \end{array}$$

or

(32) $$\begin{array}{c} g\left(\left|G_{\sigma_{1}}\cdot \nabla u_{k}\left(x,y,t\right)\right|\right)=\frac{1}{1+{\left(\left|G_{\sigma_{1}}\cdot \nabla u_{k}\left(x,y,t\right)\right|/\sigma \right)}^{2}}, \end{array}$$

where |·| is the absolute value of the number and the constant *σ* controls the sensitivity to edges. However, half-point difference for $\vec{\nabla }u(i,j)$ defined in (24) cannot be computed directly. Thus second-order integral point difference can be used to approximate the one-order half-point difference. Equations (31)-(32) become

(33) g(|Gσ1·∇uk(x,y,t)|)  =e−(|Gσ1·∇uk(x,y,t)|/σ)2  ≈e−(|Gσ1·∇2uk(x,y,t)|/σ)2,

(34) g(|Gσ1·∇uk(x,y,t)|)  =11+(|Gσ1·∇uk(x,y,t)|/σ)2  ≈11+(|Gσ1·∇2uk(x,y,t)|/σ)2.

The new AD based on the enclosed second-order difference is defined as

(35) ∂u(i,j,t)∂t =div⁡(g0∇0u(i,j,t)g1∇1u(i,j,t)g2∇2u(i,j,t)⋮g(2n+1)2−3∇(2n+1)2−3u(i,j,t)g(2n+1)2−2∇(2n+1)2−2u(i,j,t)g(2n+1)2−1∇(2n+1)2−1u(i,j,t)),

where the ∇_*k*_ *u*(*i*, *j*, *t*), *k* = 0,…, (2*n* + 1)^2^ − 1, are the components of vector $\vec{\nabla }u(i,j,t)$ in (24) and *g*_*k*_, *k* = 0,…, (2*n* + 1)^2^ − 1, defined in (30) are the components of $\vec{g}$ in (29). Moreover, the numerator of *g*_*k*_ in (30) can be approximated by (33) or (34).

The above equation can be represented as

(36) $$\begin{array}{c} \frac{\partial u\left(i,j,t\right)}{\partial t}=\sum_{k=0}^{{\left(2n+1\right)}^{2}-1}g_{k}\nabla_{k}^{2}u\left(i,j,t\right), \end{array}$$

where ∑_*k*=0_^(2*n*+1)^2^−1^ *g*_*k*_ = 1 and the right-hand side of (36) is the normalized weight ELO. ∇_*k*_^2^ *u*(*i*, *j*, *t*) is the second-order difference of the *k*th components of *u*(*i*, *j*) which can be computed according to (26).

Thus the explicit form for solving (36) is

(37) u(i,j,t+1)=u(i,j,t)+λ∑k=0(2n+1)2−1gk∇k2u(i,j,t),

where *u*(*i*, *j*, *t* + 1) is the gray level of (*i*, *j*) at time *t* + 1 and *g*_*k*_, ∇_*k*_^2^ *u*(*i*, *j*, *t*) are the same as in (36).

## 4. Experiments and Discussion

The main objective for smoothing LDCT images is to delete the noise while preserving anatomy details for the images. Experiments in this section are designed to check performance of different AD methods in LDCT imaging which used both real LDCT sinogram and standard-dose CT (SDCT) sinogram, where sinogram of SDCT will provide visual reference for recovered LDCT images.

### 4.1. Data

**Figure 2:**
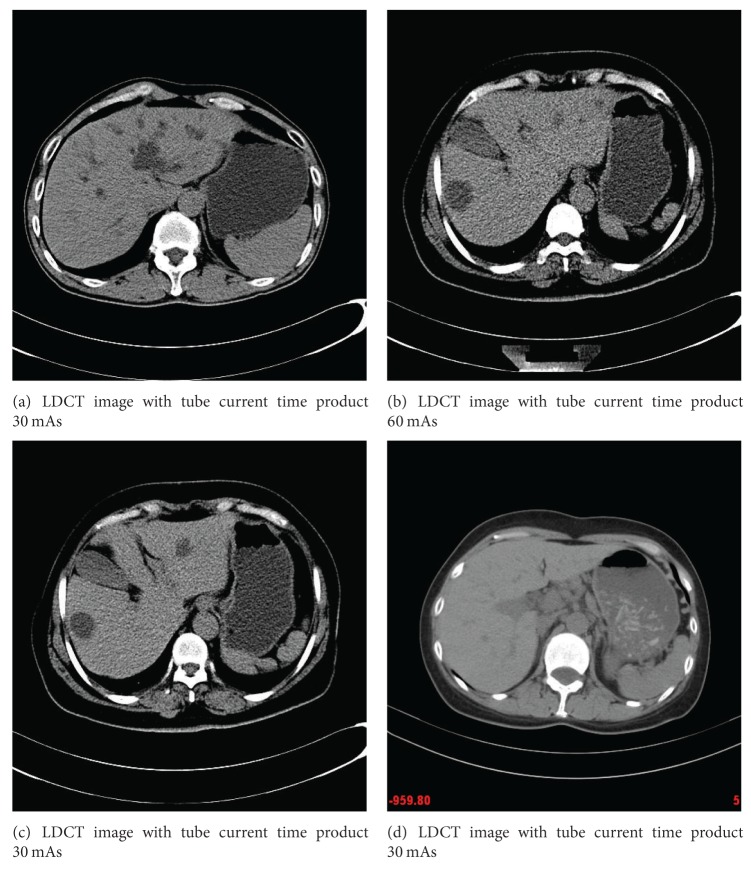
LDCT images.

**Figure 3:**
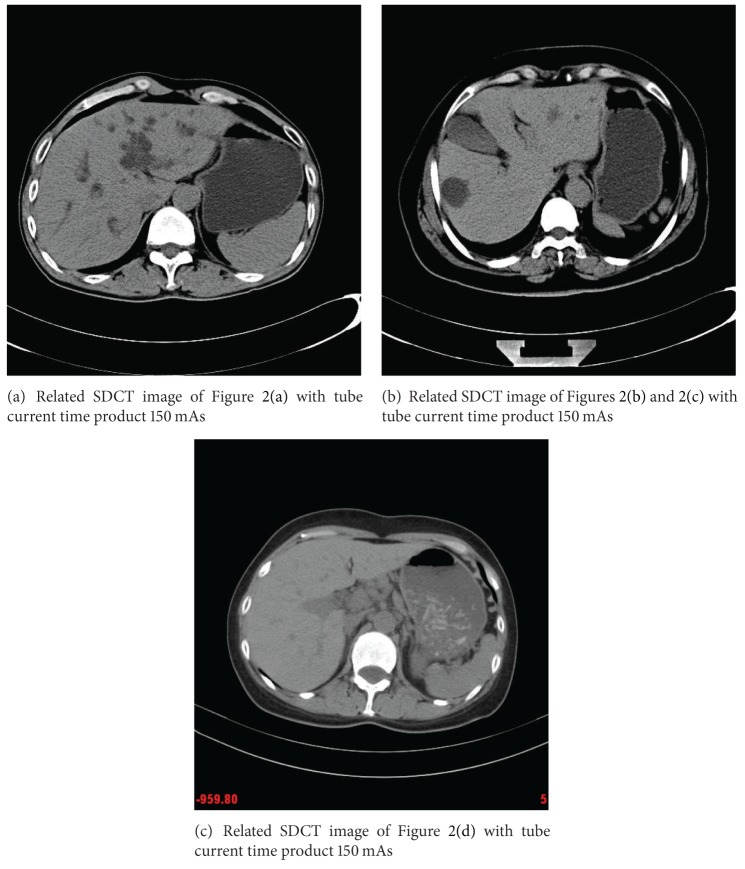
Related SDCT images.

Three abdominal CT images with different doses were scanned from a 16-multidetector row CT unit (Somatom Sensation 16; Siemens Medical Solutions) using 120 kVp and 5 mm slice thickness (Figure 2). Other remaining scanning parameters are gantry rotation time, 0.5 second; detector configuration (number of detector rows section thickness), 16 × 1.5 mm; table feed per gantry rotation, 24 mm; pitch, 1 : 1; and reconstruction method, Filtered Back Projection (FBP) algorithm with the soft-tissue convolution kernel “B30f." Different CT doses were controlled by using two different fixed tube current 30 mAs and 150 mAs (or 60 mA and 300 mAs) for LDCT and standard-dose CT (SDCT) protocols, respectively (see Figures 2 and 3). The CT dose index volume (CTDIvol) for LDCT images and SDCT images is in positive linear correlation to the tube current and is calculated to be approximately ranged from 15.32 mGy to 3.16 mGy [18].

### 4.2. Compared Methods

PM and RPM have been discussed in detail in Section 2. In this subsection, we will briefly introduce two new AD methods in [3, 11].

In [3], the authors propose a new method to make AD well posed by posing edge constraint. That is, edge is detected by canny operator to form an edge map; then a Gaussian smoothing is carried on the edge map to design a fuzzy edge function; finally, 1 is subtracted by the fuzzy edge function to get *α*(*x*, *y*, *t*). Therefore, the diffusion equation becomes

(38) $$\begin{array}{c} \frac{\partial u\left(x,y,t\right)}{\partial t}=\mathrm{div}\left(\alpha \left(x,y,t\right)g\left(x,y,t\right)\nabla u\left(x,y,t\right)\right). \end{array}$$

The authors of [11] propose a hybrid scheme that combines forward and backward differences with AD by tensor. The tensors obtained by our approach depend on four directional derivatives of the intensity of an image, and hence they are adaptively determined by local image structure. The proposed diffusion filter is isotropic in the interior of a region, whereas it is anisotropic at edges.

### 4.3. The Parameter Choice

 There are five methods will be compared in this paper: PM, RPM, the method proposed in [3] which is named as well-posed PM (WE-PM), the method proposed in [11] which is named as new tensor PM (WT-PM), and our method (RMDMS-AD). In order to ensure that comparison is put on a fair level, the common used parameters are set to the same value.

The common used parameters for five methods include gradient modulus threshold *σ* that controls the conduction, integration constant *λ* (0 ≤ *λ* ≤ 1/7), and iteration number *t*.

Due to numerical stability, *λ* is set to its maximum value 1/7.

Intuitively, the bigger the *σ* is, the smoother the recovered image is. Therefore, *σ* should be set to a small number. In this paper, it is set to 2 to preserve EADs.

The iteration number *t* is very important in AD. That is, too big *t* will make over-smooth image while too small *t* will still leave many noises. In order to study the performance of five compared methods with different iteration numbers *t* and fixed other parameters, *t* is set to 3, 5, 10, and 20, respectively.

The standard deviation of smoothed Gaussian kernel for the image *σ*_1_ used for RPM, WT-RPM, and RMDMS-AD is also set to 1.5 since, in [3], the authors suggest that *σ*_1_ should be a small number.

Another parameter is the number of scale *s*, which is very important to support the start point of our method where the big scale will have better performance than the small scale. Thus, the *s* is set to 1, 2, and 3 to study the different behavior in different scales.

### 4.4. Results and Discussion

**Figure 4:**
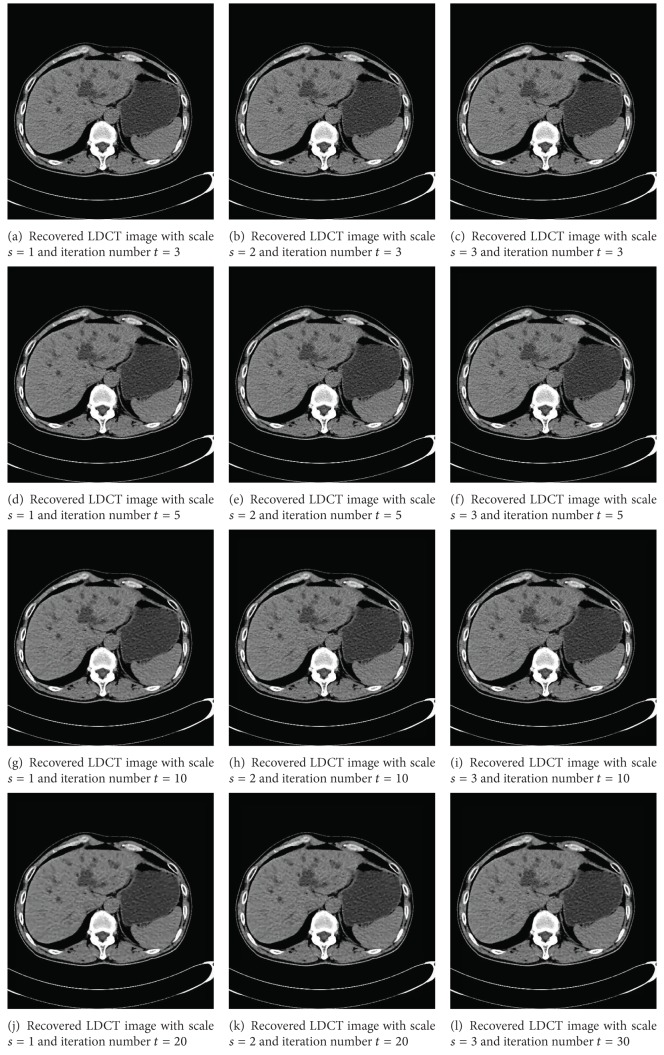
Recovered LDCT images of Figure 2(a) using RMDMS-AD with different scales *s* and different iteration numbers *t*. The first column: *s* = 1; the second column: *s* = 2; the three column: *s* = 3. The first row: *t* = 3; the second row: *t* = 5; the third row: *t* = 10; the fourth row: *t* = 30.

**Figure 5:**
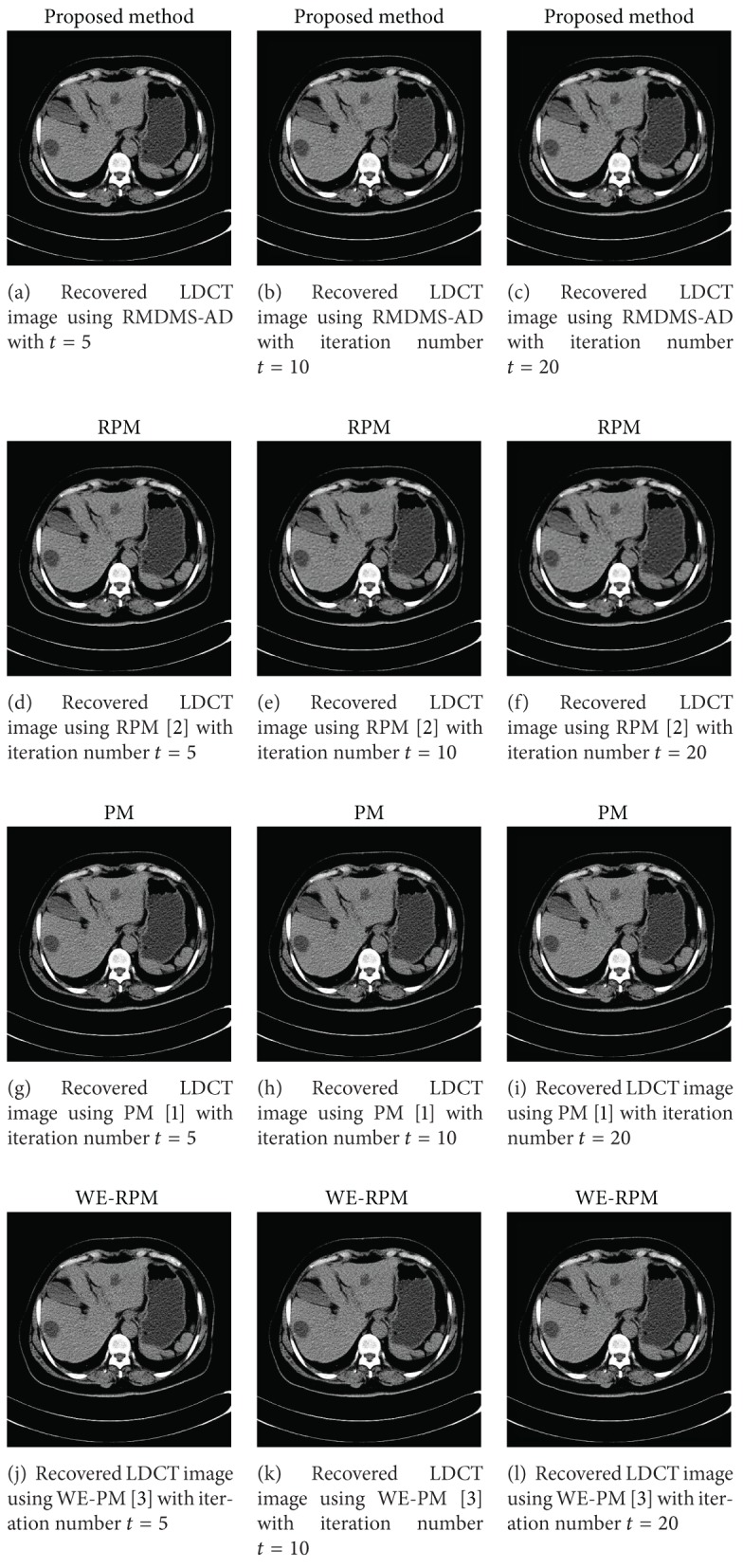

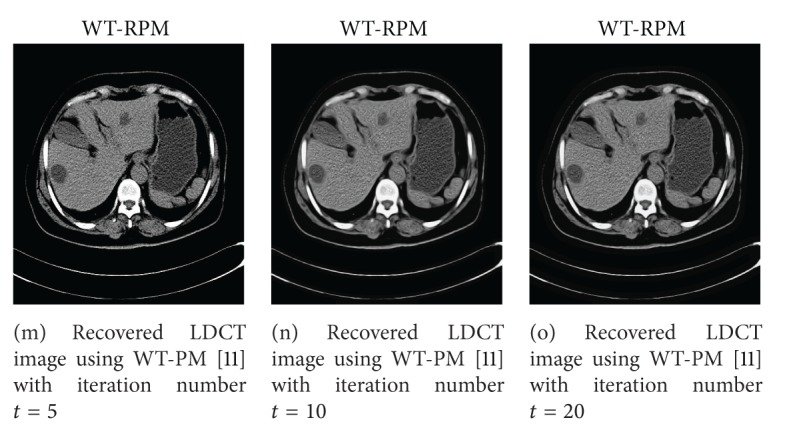
Recovered LDCT images of Figure 2(c) using RMDMS-AD (the first row), RPM (the second row [2]), PM (the third row [1]), WE-PM (the fourth row [3]), and WT-PM (the fifth row [11]) with different iteration numbers *t* = 5 (the first column), *t* = 10 (the second column), and *t* = 20 (the third column). The other parameters include *σ*_1_ = 1.5, *σ* = 2, and *λ* = 1/7 and scale *s* = 3 for the proposed method.

From Figure 4, noises in all reconstructed images are suppressed in different extent and EADs in these methods are preserved, which show that the proposed method is a promising LDCT imaging way. It also can be seen that the multiscale has better performance in preserving EADs. That is, from left to right of each row much more EADs are preserved. In addition, we also can observe that big *t* leads to over-smooth image. Thus, from top to down of each column in Figure 4, the smoother and smoother images can be obtained. Like most of existing AD methods, iteration numbers for the proposed scheme should be chosen carefully. Therefore, we will compare performance of comparison methods on Section 4.2 with *t* = 5,10,20 (see Figure 5). The reconstructed image of WT-PM has many odd artifacts from *t* = 5 to *t* = 20. These artifacts will lead to error diagnosis. Thus WT-PM does not suit for LDCT imaging.

In Figure 5 the reconstructed LDCT images for PM (the third row of Figure 5 [1]) and WE-PM (the fourth row of Figure 5 [3]) left so many noises even after 20 times of iteration (the last column of related row). The other reconstructed LDCT images also have similar situation, which is caused by the illness of AD equation and serious noise for LDCT.

RPM can be considered as a special case of the proposed method. That is, when scale *s* = 1, RMDMS-AD becomes RPM. Just as discussed in this section, RMDMS-AD has better performance than RPM. Thus RMDMS-AD can preserve more EADs for LDCT, which is very important in clinical diagnosis. RPM and RMDMS-AD can suppress noise greatly which remind us that “regularization” is an essential technology in AD. 

## 5. Conclusions

In this paper, we propose a new AD for LDCT sinogram imaging using multiscales and multidirections, named RMDMS-AD. Since not only multidirections can reduce discrete error greatly but also regularization and multiscales can provide big searching regions for solutions of AD equation, the new proposed method has good performance both in noise suppressing and DsGN preserving. Moreover, RMDMS-AD does not produce new artifacts in denoising which is very important in clinical diagnosis.
